# Antioxidant effects of vitamins in type 2 diabetes: a meta-analysis of randomized controlled trials

**DOI:** 10.1186/s13098-018-0318-5

**Published:** 2018-03-14

**Authors:** Maria E. Balbi, Fernanda S. Tonin, Antonio M. Mendes, Helena H. Borba, Astrid Wiens, Fernando Fernandez-Llimos, Roberto Pontarolo

**Affiliations:** 1 Pharmaceutical Sciences Postgraduate Programme, Universidade Federal do Paraná, Curitiba, Brazil; 2 Department of Pharmacy, Pharmaceutical Sciences Postgraduate Program, Universidade Federal do Paraná, Av. Prof. Lothario Meissner 632, Curitiba, 80210-170 Brazil; 3 Research Institute for Medicines (iMed.ULisboa), Department of Social Pharmacy, Faculty of Pharmacy, Universidade de Lisboa, Lisbon, Portugal

**Keywords:** Diabetes mellitus, Antioxidant capacity, Systematic review, Vitamin

## Abstract

**Background:**

Vitamins are essential micronutrients with antioxidant potential that may provide a complementary treatment for patients with chronic diseases. Our aim was to assess the effect of vitamin supplementation on the antioxidant status and glycemic index of type 2 diabetes mellitus patients.

**Methods:**

We performed a systematic review with meta-analyses. Electronic searches were conducted in PubMed, Scopus, and Web of Science (December 2017). Randomized controlled trials evaluating the effect of any vitamin or vitamin complex supplementation on antioxidant status as primary outcome were included. The outcomes considered were: reduction of malondialdehyde (MDA); augmentation of glutathione peroxidase (GPx); changes in total antioxidant capacity (TAC), enhance in superoxide dismutase enzyme—SOD, and thiobarbituric acid reactive substances (TBARS). Outcomes of glycemic control were also evaluated. Pairwise meta-analyses were performed using software Review Manager 5.3.

**Results:**

Thirty trials fulfilled the inclusion criteria, but only 12 could be included in the meta-analyses of antioxidant outcomes. The most commonly studied vitamins were B, C, D and E. Vitamin E was related to significant reduction of blood glucose as well as glycated hemoglobin compared to placebo, while both vitamins C and E were mainly associated with reducing MDA and TBARS and elevating GPx, SOD and TAC, compared to placebo. However, outcome reports in this field are still inconsistent (e.g. because of a lack of standard measures).

**Conclusions:**

Supplementation of vitamin E may be a valuable strategy for controlling diabetes complications and enhancing antioxidant capacity. The effects of other micronutrients should be further investigated in larger and well-designed trials to properly place these complementary therapies in clinical practice.

**Electronic supplementary material:**

The online version of this article (10.1186/s13098-018-0318-5) contains supplementary material, which is available to authorized users.

## Background

Diabetes mellitus (DM) is a chronic metabolic disorder characterized by an increase in blood glucose concentration (fasting blood glucose ≥ 126 mg/dL). There are currently 425 million people with diabetes worldwide, and this number is expected to reach 629 million by 2045, with type 2 diabetes (T2DM) being the most expressive form of the disease [1, 2]. The American Diabetes Association (ADA) and the European Association for the Study of Diabetes (EASD) consensus statement on the management of T2DM recommend life-style changes (healthy diet and physical activity) in combination with metformin at the time of diagnosis, and the addition of other medication in patients who do not achieve the desired glycemic control [1]. Lowering glycated hemoglobin (HbA1c) to below 7% has been shown to be one of the primary endpoints in reducing microvascular complications of DM and possibly macrovascular disease [3].

Current evidence has demonstrated that oxidative stress plays an important role in the pathogenesis of chronic diseases such as DM [4, 5] and may diminish the antioxidative defense system of the body, increasing the oxidative load [6]. Some studies have shown that individuals with low concentrations of antioxidants are at increased risk of diabetes complications [5, 7, 8] and that T2DM is associated with endothelial dysfunction [9]. These conditions may develop into macro and microvascular diseases such as retinopathy, nephropathy, lower extremity amputations, coronary artery and cardiovascular diseases [10–12], which are the main causes of morbidity and mortality worldwide [2].

The damaging effects of oxidative stress are mainly caused by the production of free radicals of oxygen and reactive oxygen species (ROS), but these substances can be modified by enzymatic or non-enzymatic antioxidants such as superoxide dismutase, vitamins, minerals, and polyphenols [13]. A previous study described how the supplementation with multivitamins in a population with a high prevalence of micronutrient deficiency significantly decreased cerebrovascular disease mortality [14]. Other researchers have analyzed the antioxidant properties of natural products through chemical or biological methods. They have suggested that the consumption of food rich in antioxidants can retard or prevent the occurrence of disease [15, 16]. Nevertheless, previous systematic reviews and individual randomized controlled trials (RCTs) that have measured the effect of vitamin supplementation on antioxidant status and glycemic control of diabetic patients have provided conflicting results, so that the benefit, or otherwise, of such supplementation remains uncertain [17–21].

Thus, we aimed to conduct a systematic review and pairwise meta-analyses to gather current evidence on the effects of any vitamin supplementation on antioxidant status in T2DM patients, in order to elucidate its real benefits.

## Methods

We conducted and reported this systematic review and meta-analyses according to the Cochrane Recommendations and PRISMA (Preferred Reporting Items for Systematic Reviews and Meta-Analyses) guidelines [22, 23]. Two independent reviewers performed all the steps and discrepancies were solved by consensus with a third author.

### Search strategies and inclusion criteria

We searched for relevant articles in the databases PubMed, Scopus and Web of Science, without any time limit (updated December 18th, 2017). In addition, we conducted a manual search on the reference lists of the retrieved articles, reviews and trial registration databases to identify registers missed by the electronic search. Complete search strategies are presented in Additional file 1: Search strategies.

We included RCTs assessing adult patients (over 18 years old) of any gender with any stage of T2DM and evaluating plasmatic antioxidant parameters or oxidative stress. Patients received vitamins (types A and/or B complex and/or C and/or D and/or E or variants administered alone or in combination with other vitamins, micronutrients or minerals) irrespective of form, dosage, duration or route of administration compared with placebo or no treatment or other vitamins (active control).

Two researchers independently screened titles and abstracts of the articles retrieved by the systematic review to identify irrelevant records. In a second stage, full text articles were evaluated to identify any of the following exclusion criteria: non-randomized controlled trials (type of studies); interventions other than vitamins; individuals aged under 18 years; different populations or other type of diabetes (prediabetes, type 1 diabetes mellitus, gestational diabetes mellitus); outcomes measure other than antioxidant-related; trials published in non-roman characters.

### Data extraction and quality assessment

The following data were independently extracted from the included studies by two researchers: baseline characteristics (authors’ names, year of publication, study design, country, sample size, gender, age, patients’ condition, trial duration); methodological aspects; and clinical outcomes of interest. For primary outcomes, studies should report alterations in plasma antioxidant parameters or oxidative stress, such: vitamin levels, antioxidant enzyme levels [superoxide dismutase (SOD), glutathione peroxidase (GPx) and catalase (CAT)], oxidative stress biomarkers (e.g. harmful products MDA (malondialdehyde) and thiobarbituric acid reactive substances (TBARS) or changes in plasma total antioxidant capacity (TAC). Other changes in anthropometric and glycemic parameters such as fasting blood glucose and HbA1c reduction, regarded as the ‘core outcome set’ for diabetes control, were also collected, when available.

Two different instruments, the Jadad score [24] and the Cochrane Collaboration’s tool for assessing the Risk of Bias [22], were used to evaluate the included studies’ methodological aspects, such as proper randomization, blinding, account for patients withdrawals and dropouts and other bias that may affect data interpretation.

### Statistical analyses

Pairwise meta-analyses of the included RCTs were performed for the main outcome measures whenever the number of studies for each outcome of interest allowed. These analyses were conducted using the software Review Manager version 5.3 (The Nordic Cochrane Centre, The Cochrane Collaboration, Copenhagen, Denmark).

For each meta-analysis we used the random effects model and the inverse variance (IV) method to interpolate the mean differences (MD) or standardized (std.) mean differences (SMD) of each study from baseline. Results are reported with a 95% confidence interval (CI). A p value less than 0.05 (two-tailed) was considered indicative of a statistically significant difference between groups. The between-trial heterogeneity was assessed using the inconsistency index value (I^2^) (I^2^ > 50% indicates high and significant heterogeneity) [22]. We also conducted sensitivity analyses to test the robustness of the results in order to evaluate the impact of any study on data heterogeneity. The analysis consisted of the hypothetical sequential removal of studies from the meta-analysis. When possible, subgroup analyses were also performed.

## Results

**Fig. 1:**
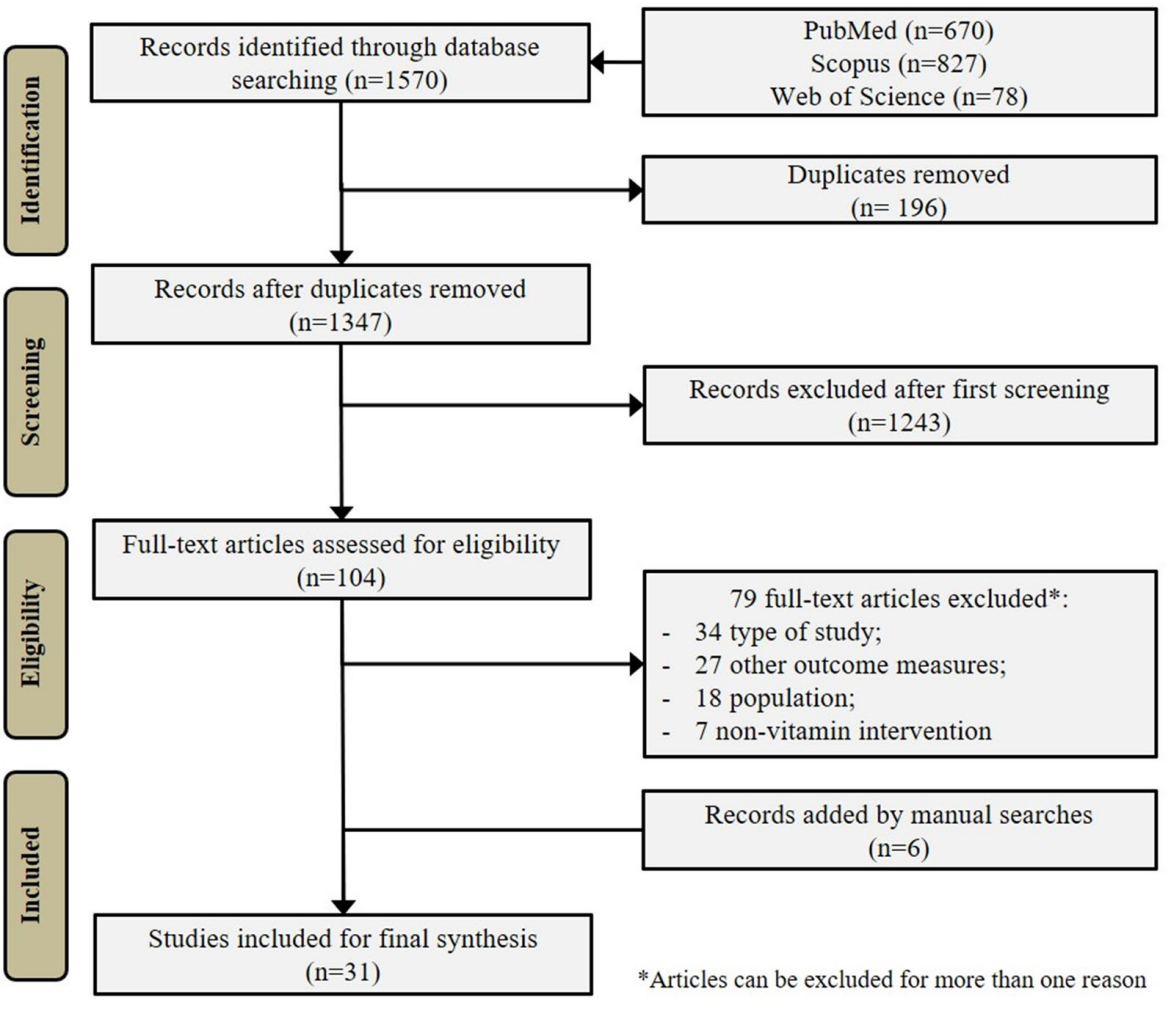
Flowchart of the systematic review process

The systematic search conducted in the three databases retrieved 1570 records and 196 were excluded as duplicates. During the study’s title and abstract reading process (screening), 1243 records were excluded and 104 were considered for full-text appraisal, of which 25 articles were suitable for final analyses. Six articles were added from manual searches, finally yielding 31 articles representing 30 RCTs [25–55] (Fig. 1). The main characteristics of the included studies are provided in Table 1.

**Table 1:** Characteristics of the included studies

Author, years	Country	Treatments	N (sample)	Duration (weeks)	Main outcome measures	Age (years)	Male (%)	Jadad score
Aghamohammadi, 2011 [25]	Iran	Vitamin B9 5 mg/day Placebo	70	8	MDA; TAC; vitamin concentration	58.7 ± 7.2 55.6 ± 9.3	100	3
Anderson, 2006 [26]	Wales	Vitamin C 1000 mg/day Placebo	20	6	TBARS	52.7 ± 6.9 53.6 ± 7.9	40.0	2
Antoniades, 2004 [27]	Greece	Vitamin C 2000 mg/day Placebo	17	4	Vitamin concentration; TNF	48.5 ± 6.6 52.6 ± 5.9	58.8	1
Ble-Castillo, 2005 [28]	United States	Vitamin E 800 IU/day Placebo	33	6	Blood glucose; HbA1c; MDA; GPx	51.3 ± 14.0 55.3 ± 11.6	0	2
Chen, 2006 [29]	United States	Vitamin C 800 mg/day Placebo	32	4	Blood glucose; vitamin concentration	50.0 ± 1.0	40.6	3
Dalan, 2016 [30]	Singapore	Vitamin D 4000 IU/day Vitamin D 2000 IU/day Placebo	64	16	HbA1c; vitamin concentration; GSH	52.2 ± 8.2 54.8 ± 10.8	51.6	5
Gariballa, 2013 [31]	Arab Emirates	Vitamin complex (B, C, E) Placebo	100	12	MDA; TNF; vitamin concentration	52 (44–56) 51 (42–60)	41.0	2
Haghighat, 2014 [32]	Iran	Vitamin E enriched canola oil 15 ml/day Placebo oil	45	8	CRP; NO	55.9 ± 5.9 55.2 ± 5.6	26.7	2
Hejazi, 2015 [33]	Iran	Vitamin E 400 IU/day Placebo	27	6	Blood glucose; MDA	48.0 ± 6.3 46.6 ± 7.6	26.0	3
Jamalan, 2015 [34]	Iran	Vitamin C 1000 mg/day Vitamin E 300 mg/day	80	4	Blood glucose; CRP; TNF	52.0 ± 8.0	100	2
Jorde, 2009 [35]	Norway	Vitamin D 40,000 IU/week Placebo	32	24	Blood glucose; HbA1c; vitamin concentration	57.7 ± 9.7 54.8 ± 5.9	56.2	2
Lai, 2008 [36]	Japan	Chromium 1000 µg Vitamin E 800 IU + chromium Placebo	30	24	Blood glucose; HbA1c; SOD; CAT; TBARS; TAS; GPx	53.2 ± 2.0 51.5 ± 1.7 50.5 ± 1.9	46.7	3
Lu, 2005 [37]	Sweden	Vitamin C 3000 mg/day Placebo	20	2	Blood glucose; HbA1c; IL-6	–	60.0	2
Mahmoudabadi, 2014 [38]	Iran	Eicosapentaenoic acid 500 mg/day Vitamin C 200 mg/day Vitamin C + eicosapentaenoic acid Placebo	81	8	Blood glucose; HbA1c; vitamin concentration; SOD; MDA; TAC; GPx	54.0 ± 5.0 53.0 ± 5.0 52.0 ± 6.0 50.0 ± 8.0	100	2
Manzella, 2001 [39]	Italy	Vitamin E 600 mg/day Placebo	50	16	HbA1c; TBARS	64.3 ± 4.7 65.1 ± 3.9	–	3
Mason, 2016 [40]	Australia	Vitamin C 1,000 mg/day Placebo	13	16	Insulin; GSH; IL-6	59.4 ± 3.5	92.3	3
Mazloom, 2011 [41]	Iran	Vitamin C 1,000 mg/day Placebo	27	6	Plasma lipid parameters; MDA	47.0 ± 8.9 46.6 ± 7.6	42.1	2
Mullan, 2002 [42]	United Kingdom	Vitamin C 500 mg/day Placebo	30	4	Plasma lipid parameters; LDL-ox	61.0 ± 6.5 57.9 ± 6.6	73.4	3
Nikooyeh, 2011/2014 [43, 44]	Iran	Yogurt (150 mg calcium/250 mL) Vitamin D fortified (150 mg calcium + 500 IU/250 mL) Vitamin D fortified (250 mg of calcium + 500 IU/250 mL) *Two bottles/day = 500 mL/day	90	12	Blood glucose; HbA1c; vitamin concentration; SOD; MDA	50.8 ± 6.6 51.4 ± 5.4 49.9 ± 6.2	38.9	2
Paolisso, 2000 [45]	Italy	Vitamin E 600 mg/day Placebo	40	8	TBARS; vitamin concentration	58.3 ± 6.4 56.7 ± 5.3	52.5	3
Park, 2002 [46]	Korea	Vitamin E 200 mg/day Placebo	98	8	Blood glucose; HbA1c; SOD; CAT	49.4 ± 9.3 49.5 ± 10.1	59.2	2
Reaven, 1995 [47]	United States	Vitamin E 1600 lU/day Placebo	21	10	Blood glucose; HbA1c; LDL-ox	60.8 ± 6.1 61.8 ± 8.4	100	2
Shab-Bidar, 2015 [48]	Iran	Vitamin D fortified Yogurt 500 UI, 500 ml/day Yogurt	100	12	Blood glucose; HbA1c; SOD; MDA; TAC; GSH	52.6 ± 6.3 52.4 ± 8.4	43.0	3
Sugden 2007 [49]	United Kingdom	Vitamin D 100,000 UI/day Placebo	34	8	HbA1c; vitamin concentration; NO	64.9 ± 10.3 63.5 ± 9.5	52.9	5
Tessier, 2009 [50]	Canada	Vitamin C 500 mg/day Vitamin C 1000 mg/day Placebo	36	12	Plasma lipid parameters; GSH	72.0 ± 5.0 72.0 ± 4.0 71.0 ± 4.0	22.2	3
Vafa, 2015 [51]	Iran	Vitamin E enriched canola oil 15 ml/day Canola oil	45	8	Blood glucose; MDA; TAC	55.9 ± 5.9 55.2 ± 5.6	73.3	4
Winterbone, 2007 [52]	United Kingdom	Vitamin E 1200 IU α-tocopherol/day Placebo	19	4	Blood glucose; insulin; vitamin concentration	62.7 ± 1.8 61.9 ± 1.9	100	2
Witham, 2010 [53]	United Kingdom	Vitamin D3 100,000 IU/day Vitamin D3 200,000 IU/day Placebo	41	16	HbA1c; vitamin concentration; GSH	65.3 ± 11.1 63.3 ± 9.6 66.7 ± 9.7	67.2	3
Wu, 2007 [54]	Australia	Vitamin E α-tocopherol 500 mg/day Vitamin E mixed tocopherols 500 mg/day Placebo	55	6	SOD; GPx	64.0 ± 7.0 58.0 ± 4.0 62.0 ± 7.0	74.5	2
Yiu, 2013 [55]	China	Vitamin D 500 IU/day Placebo	100	12	Blood glucose; HbA1c; SOD	65.8 ± 7.3 64.9 ± 8.9	50.0	4

*CAT* catalase; *CRP C*-reactive protein; *GPx* glutathione peroxidase; *GSH* glutathione; *HbA1c* glycated hemoglobin; *IL*-*6* interleukin; *LDL*-*ox* oxidized low-density lipoprotein; *MDA* malondialdehyde; *NO* nitric oxide; *SOD* superoxide dismutase; *TAC* total antioxidant capacity; *TBARS* thiobarbituric acid reactive substances; *TNF* tumor necrosis factor

All studies involved patients diagnosed with T2DM (n = 1430) and were conducted mainly in Iran (n = 9 trials) [25, 32–34, 38, 41, 43, 44, 48, 51]; followed by the United Kingdom (n = 4) [42, 49, 52, 53] and the United States of America (n = 3) [28, 29, 47]. Evaluated treatments comprised: vitamin B (n = 1 study) [25], vitamin C (n = 10) [26, 27, 29, 34, 37, 38, 40–42, 50]; vitamin D (n = 7) [30, 35, 43, 44, 48, 49, 53, 55]; vitamin E (n = 12) [28, 32–34, 36, 39, 45–47, 51, 52, 54], and a mixture of vitamins B, C and E (n = 1) [31]. In four of these trials (13.3%), vitamins were delivered by food fortification (oil or yogurt) [32, 44, 48, 51]. A placebo or negative control was the main comparator in 29 studies (96.7%), while eight trials (26.7%) included head-to-head comparisons. Duration of treatment ranged from two to 24 weeks and patients’ age ranged from 46 to 72 years.

Overall, the methodological quality of included trials was low to moderate, with a mean Jadad Score of 2.7 (range 1–5). All studies scored on randomization, but only 20% of them properly described how randomization was achieved. Almost all trials (90%) accounted for patient’ withdrawals or dropouts, and half of the studies was double-blinded. However, only two trials described the blinding methods. The Risk of Bias assessment (see Additional file 1: Quality assessment), established that trials were of low risk of bias (> 75%) in the domains of randomization, incomplete outcome data and selective reporting. Allocation concealment was considered unclear in 25 trials (86.2%), and studies often failed to provide details regarding blinding of participants or outcome measures. Overall, 70% of trials were funded by industries or reported conflict of interest.

Considering the primary outcomes of interest related to antioxidant status, 12 RCTs (encompassing 13 articles) were able to be included in the meta- analyses (all of them compared vitamin to placebo) [25, 28, 33, 36, 38, 39, 41, 43–45, 48, 51, 54]. Not all studies were statistically evaluated since outcomes were not comparable (e.g. because of lack of raw data). Gathering evidence, especially on antioxidant potential, was hampered by the lack of standardization of outcome reporting in the clinical trials (e.g. inconsistent reporting, using different measures, scales and units).

**Fig. 2:**
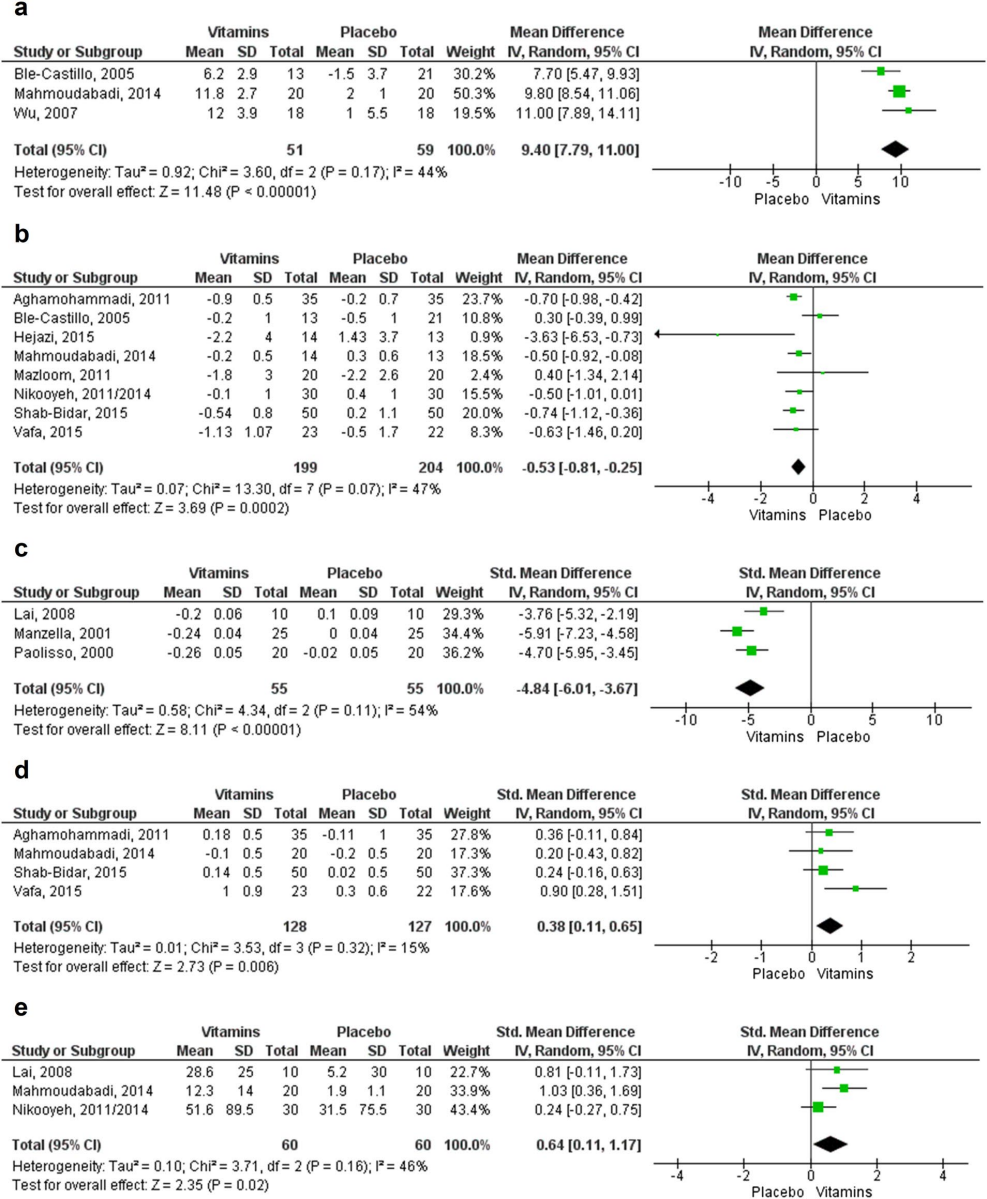
Forest plots for the outcomes of antioxidant status. **a** Augmentation of GPx level (U/g Hb). **b** Reduction of MDA (nmol/L). **c** Reduction of TBARS (µmol/L). **d** Changes in TAC (mmol/L). **e** Changes in SOD (U/g). Statistical method: Mean difference (MD) and Std. Mean Difference (SMD), IV, Random, 95% confidence interval

Meta-analyses were obtained for augmentation of GPx levels (Units/gram of Hemoglobin—U/g Hb), reduction in plasma MDA (nmol/L) and TBARS (µmol/L) reductions, and favorable changes in TAC (mmol/L) and SOD (U/g Hb). In these cases, no subgroup analyses were performed due to the limited number of studies. Overall, results were statistically different from placebo to favored the use of vitamins with values of MD 9.40 (95% CI [7.79; 11.00]) for GPx (p < 0.001) and MD − 0.53 (95% CI [− 0.81; − 0.25]) for MDA (p < 0.001), with I^2^ values of 44 and 47%, respectively. Vitamins were also superior to placebo in reducing TBARS with an overall effect size of SMD − 4.84 (95% CI [− 6.01; − 3.67]) (p < 0.001; I^2^ = 54%) and in increasing TAC (SMD 0.38 [0.11; 0.65]; p = 0.006) and SOD levels (SMD 0.64 [0.11; 1.17]; p = 0.02). These positive results came mostly from studies where the interventions were vitamin E (n = 7 trials) [28, 33, 36, 39, 45, 51, 54]; vitamin C (n = 2) [38, 41] and vitamin D (n = 2) [44, 48] (see Fig. 2).

**Fig. 3:**
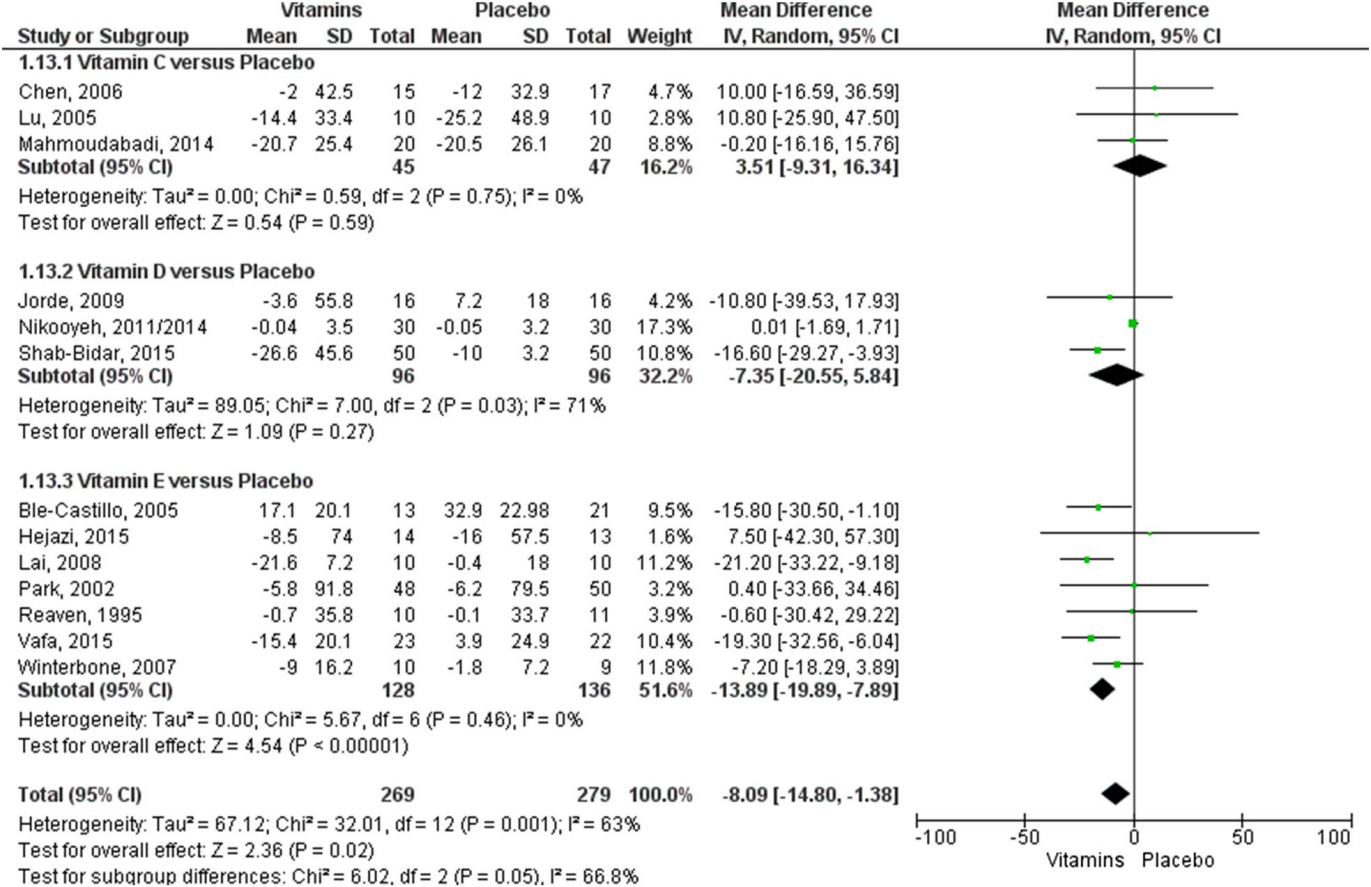
Forest plot for the outcome measure of blood glucose mean change from baseline (mg/dL). Statistical method: Mean difference (MD), IV, Random, 95% confidence interval

**Fig. 4:**
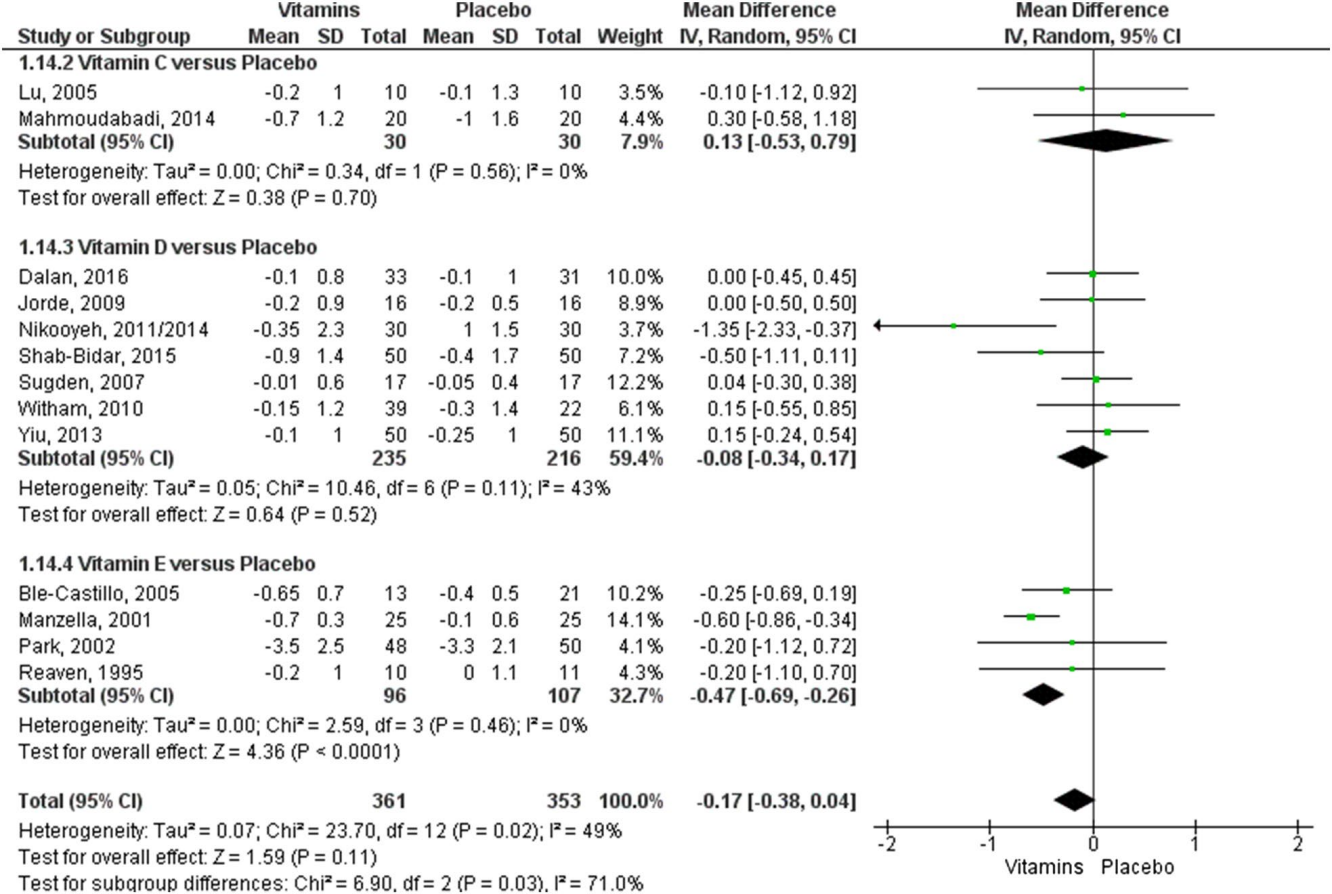
Forest plot for the outcome measure of HbA1c mean change from baseline (%). Statistical method: Mean difference (MD), IV, Random, 95% confidence interval

The meta-analyses of the glycemic control parameters (17 included trials obtained from 18 articles) are shown in Figs. 3, 4 [28–30, 33, 35–39, 43, 44, 46–49, 52, 53, 55]. No statistical differences were observed in subgroup analyses comparing vitamins C or D with placebo. However, for both outcomes of mean change in blood glucose (mg/dL) and reduction of HbA1c (as a percentage), the effects of vitamin E were significantly better than the control (values of MD − 13.89 (95% CI [− 19.89; − 7.89]) and MD − 0.47 (95% CI [− 0.69; − 0.26]), respectively).

The moderate to high heterogeneity of some meta-analyses (I^2^ ranging from 15 to 71%) was caused by more than one study and can be considered acceptable in this context. Sensitivity analyses were conducted with all the meta-analyses (data not shown) and despite the sequential hypothetical removal of studies with reduction in the heterogeneity, the results remained unchanged.

## Discussion

Our study is the first systematic review with meta-analysis to evaluate the available evidence of vitamin supplementation in T2DM patients for the improvement of antioxidant status in different ways (GPx, SOD and TAC levels augmentation and reduction in MDA and TBARS products). Previous studies have focused on glycemic control, insulin resistance and changes in endothelial functions [18, 20, 21, 56].

Our results revealed that supplementation of certain vitamins in T2DM, especially vitamin E, can produce a significant impact on the parameters of antioxidant status and glycemic control, which may positively benefit patients. Vitamin C was more related to changes in antioxidant status, while little evidence was found for the effect of other vitamins (e.g. D or B).

The beneficial effects of vitamin E may be explained by the reduction of the damaging effects of free radicals on the structural and functional components of cells and vessel walls [56, 57]. It is believed that diabetes is associated with increased oxidative stress because of increased blood concentrations of thiobarbituric acid reactive substances and serum malondialdehyde, the end products of lipid peroxidation [58]. The adverse physiological effects which result include increased leakiness of cell membranes where the structural integrity of membranes has been altered; inactivation of membrane bound enzymes and surface receptors and the involvement of oxidized LDL (LDL-ox). When total antioxidant status (TAC) is high enough to combat the oxidative stress, the MDA and TBARS levels are in the normal limits and vice versa. Antioxidants decrease the oxidative damage directly by reacting with free radicals or indirectly by inhibiting the activity or expression of free radicals [59, 60].

Non-enzymatic antioxidants such as vitamins C and E and glutathione interrupt free radical chain reactions. The combination of these vitamins appears to be promising. Although only one RCT evaluating a mixed vitamin complex was found in our systematic review [31], a previous study reported that antioxidant combinations might be an appropriate formula for the management of diabetes [61]. A 3-month study on the supplementation of vitamins C and E showed that patients’ blood glucose decreased while SOD and glutathione levels increased [62]. Moreover, the long-term use of dietary supplements, including multivitamin or mineral complexes showed benefits in C-reactive protein, HDL cholesterol, triacylglycerides, serum homocysteine, blood pressure and incidence of diabetes [14, 63–65]. However, vitamin C alone did not present a greater profile than vitamin E.

In the literature, vitamin D is related to gene expression control which may trigger a biological response to oxidative stress, such as inhibiting nitric oxide synthase (iNOS) or increasing glutatione levels [66]. The antioxidant effect of vitamin D is among the most recent non-calcemic roles suggested for this compound [67]. There is evidence from both humans and animal models suggesting that vitamin D may play an important role in modifying the risk of diabetes [66, 68]. Low vitamin D status is associated with future macrovascular events in patients with T2DM. This association may be the result of the link between vitamin D status and the renin-angiotensin system, endothelial function, blood pressure, or even chronic inflammation [20, 69, 70]. However, our results were constrained in defining a vitamin D antioxidant profile, since few RCTs involving this micronutrient were included [43, 44].

Some trials [32, 44, 48, 51] did not use direct drug supplementation but incorporated the vitamin in food (e.g. oil, yogurt), which may have affected final results. Moreover, because the total daily dosage of vitamin intake and treatment duration varied among the studies, effects on antioxidant and glycemic profiles may have been underestimated. Regimens for vitamin C varied from 500 to 3000 mg/day; for vitamin E they ranged from 400 to 1600 IU/day and for vitamin D doses were of 500 to 200,000 IU/day. Longer period trials with reasonable lower daily doses may increase the intracellular concentration of vitamins and result in an adequate effect that should then be evaluated.

In spite of the encouraging results reported above, the small number of studies properly reporting data prevented a fully satisfactory assessment of the outcomes related to antioxidant status. Moreover, methodological aspects of the included trials demonstrated low to moderate quality, especially concerning accurate description of randomization and blinding. The moderate to high heterogeneity in some meta-analyses can be explained by the differences in the intrinsic characteristics of studies, the conduct and design of trials with low quality, the small sample sizes of some studies, patient’s conditions with possible comorbidities, different pharmacological treatments, and differences in outcome measures.

The marked heterogeneity in the outcomes reporting of oxidative stress and antioxidant capacity might be due the lack of standardization in the selection or measurement of the outcome in clinical trials. Different measures and units are usually employed (e.g., enzyme levels (catalase, superoxide dismutase); FRAP—ferric reducing ability of plasma assay; ORAC—oxygen radical absorbance capacity assay; TAS—total antioxidant status, among others) [71, 72]. This can be justified in part because of the range of substances and antioxidant components in the organism, together with the difficulty in measuring them all at once. The issue of lack of outcome standardization is common to different areas, but has been associated with a bad reporting practice—outcome switching, and hamper comparisons between interventions [73]. The development of a core outcome set for antioxidant status in chronic diseases is important for study design and could minimize bias. Measures such as TAC, TBARS and MDA could be employed as standard.

Our study has some limitations. We included RCTs with differences in methodological design and population characteristics (e.g. age, gender, disease stage and comorbidities, diabetes treatments, study duration) and none of them were sufficiently powerful due to the relatively small number of participants. There was some difficulty in finding and gathering trials of the same vitamin or vitamin complex assessing similar outcomes. We were able to statistically analyze three vitamins (C, D and E), but other micronutrients and vitamin combinations (especially vitamins C and E) should be better investigated. Subgroup meta-analyses were poorly obtained.

We strongly recommend that further well-designed, large-scale, long-term head-to-head controlled trials and meta-analyses be carried out to demonstrate the effects of individual or multivitamin supplementations on T2DM, since previous results are promising.

## Conclusions

The consumption of vitamin E (alone or in combination) promotes health benefits since it affects plasma antioxidant capacity and the concentration of enzymes and reduces MDA and TBARS levels. T2DM patients have a high risk of experiencing micro and macrovascular complications, and daily vitamin supplementation provides an alternative strategy for metabolic control, in addition to the combination of diet, exercise and medication. These substances may represent a step forward in disease management. Further studies should be conducted to strengthen this evidence, especially for defining doses and regimen of vitamin E, and support its use in daily practice.

## Additional file

**Additional file 1.** Search strategies and Quality assessment.

## Abbreviations

ADA: American Diabetes Association
DM: diabetes mellitus
EASD: European Association for the Study of Diabetes
T2DM: type 2 diabetes mellitus
HbA1c: glycated hemoglobin
CAT: catalase
MDA: malondialdehyde
GPx: glutathione peroxidase
TAC: total antioxidant capacity
SOD: superoxide dismutase enzyme
TBARS: thiobarbituric acid reactive substances
ROS: reactive oxygen species
RCT: randomized controlled trials
