# Supplementary material for: Antioxidant effects of vitamins in type 2 diabetes: a meta-analysis of randomized controlled trials
Source: Diabetol Metab Syndr. 2018 Mar 14;10:18. doi: 10.1186/s13098-018-0318-5 (PMC5853104; doi:10.1186/s13098-018-0318-5)
Supplement: Supplementary file 1 — Additional file 1. Search strategies and Quality assessment. [file 13098_2018_318_MOESM1_ESM.docx]

**Search strategies**

| PubMed | #1 (clinical[Title/Abstract] AND trial[Title/Abstract]) OR “clinical trials”[MeSH Terms] OR “clinical trial”[Publication Type] OR random*[Title/Abstract] OR “random allocation”[MeSH Terms] OR “therapeutic use”[MeSH Subheading]  #2  "Diabetes Mellitus"[MeSH Terms] OR diabetes[Title/Abstract] OR diabetic*[Title/Abstract] OR "Diabetes Mellitus"[Title/Abstract]  #3 antioxidant*[Title/Abstract] OR "oxidation inhibitor"[Title/Abstract] OR "oxidation prevention"[Title/Abstract] OR "endothelial function”[Title/Abstract] OR "antioxidizing agent"[Title/Abstract]  #4 vitamin[Title/Abstract] OR vitamins[MeSH Terms]  #1 AND #2 AND #3 AND #4 |
| --- | --- |
| Scopus | ( TITLE-ABS-KEY (antioxidant)) AND ( TITLE-ABS-KEY ( diabetes)) AND ("clinical trial" AND random* ) AND ( TITLE-ABS-KEY (vitamin)) |
| Web of Science | (diabetes) AND Tópico: (antioxidant) AND Tópico: (vitamin) AND Tópico: ("clinical trial") |

**Quality assessment**


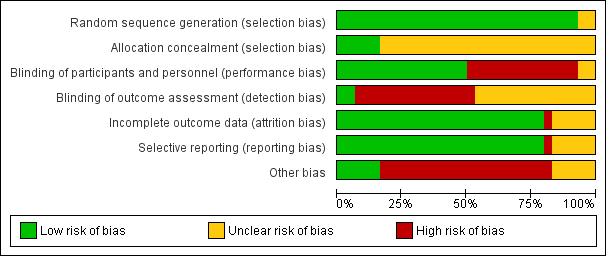


**Risk of bias graph: review authors' judgements about each risk of bias item presented as percentages across all included studies**


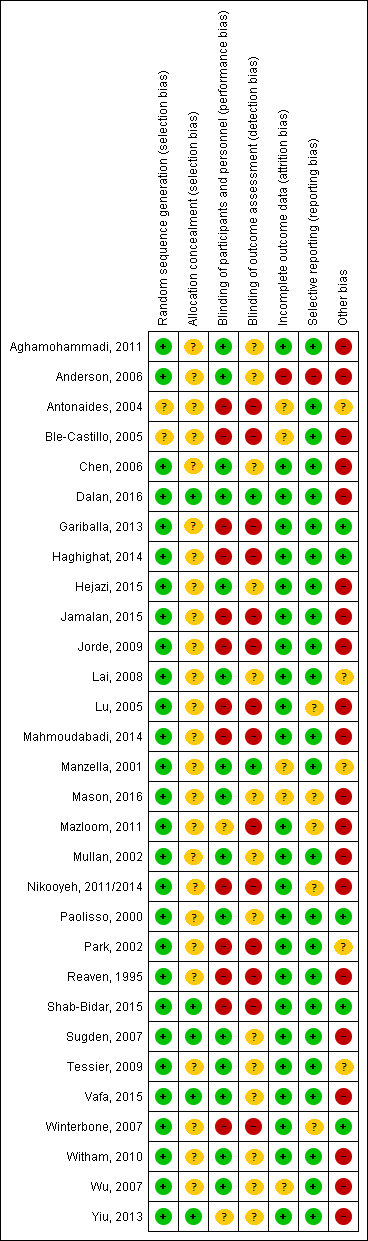


**Risk of bias summary: review authors' judgements about each risk of bias item for each included study**
